# HDL and glucose metabolism: current evidence and therapeutic potential

**DOI:** 10.3389/fphar.2015.00258

**Published:** 2015-10-31

**Authors:** Andrew L. Siebel, Sarah Elizabeth Heywood, Bronwyn A. Kingwell

**Affiliations:** Metabolic and Vascular Physiology Laboratory, Baker IDI Heart and Diabetes Institute, Melbourne, VIC, Australia

**Keywords:** HDL, glucose uptake, insulin sensitivity, insulin secretion, glucose metabolism, apolipoprotein A-I, cholesterol efflux

## Abstract

High-density lipoprotein (HDL) and its principal apolipoprotein A-I (ApoA-I) have now been convincingly shown to influence glucose metabolism through multiple mechanisms. The key clinically relevant observations are that both acute HDL elevation via short-term reconstituted HDL (rHDL) infusion and chronically raising HDL via a cholesteryl ester transfer protein (CETP) inhibitor reduce blood glucose in individuals with type 2 diabetes mellitus (T2DM). HDL may mediate effects on glucose metabolism through actions in multiple organs (e.g., pancreas, skeletal muscle, heart, adipose, liver, brain) by three distinct mechanisms: (i) Insulin secretion from pancreatic beta cells, (ii) Insulin-independent glucose uptake, (iii) Insulin sensitivity. The molecular mechanisms appear to involve both direct HDL signaling actions as well as effects secondary to lipid removal from cells. The implications of glucoregulatory mechanisms linked to HDL extend from glycemic control to potential anti-ischemic actions via increased tissue glucose uptake and utilization. Such effects not only have implications for the prevention and management of diabetes, but also for ischemic vascular diseases including angina pectoris, intermittent claudication, cerebral ischemia and even some forms of dementia. This review will discuss the growing evidence for a role of HDL in glucose metabolism and outline related potential for HDL therapies.

## Introduction

There is accumulating experimental and clinical evidence that HDL particles can control glucose metabolism via a variety of mechanisms (Drew et al., 2012; von Eckardstein and Widmann, 2014). At a mechanistic level HDL particles can alter multiple aspects of glucose metabolism. These include insulin secretion from pancreatic beta cells, and both insulin-dependent and insulin-independent glucose uptake and utilization in tissues. Some of these effects are mediated by direct signaling actions of HDL and its components, whilst others appear to be secondary to other known HDL functions, including tissue lipid efflux.

The action of HDL to move glucose from the circulation and into tissues has potential clinical relevance in terms of both reducing vascular complications by removing excess glucose from the circulation in the setting of T2DM, and also in providing adequate glucose to tissues for energy production, particularly in the context of ischemia. HDL therapeutics may therefore have application not only for the prevention and management of T2DM (Drew et al., 2012; von Eckardstein and Widmann, 2014), but also for ischemic vascular diseases including angina pectoris, intermittent claudication, cerebrovascular disease (Kingwell and Chapman, 2013) and even some forms of dementia (Stukas et al., 2014b).

This review will discuss the growing evidence for a role of HDL in glucose metabolism in the context of the evolving HDL hypothesis. It is now recognized that the main clinical variable used to assess HDL, HDL cholesterol, fails to provide an accurate representation of the multiple functions of heterogeneous HDL particles, which are composed of many hundreds of lipids and multiple proteins (Khera et al., 2011; Kingwell et al., 2014; Rohatgi et al., 2014; Saleheen et al., 2015). The role of HDL in glucose metabolism will be explored from the perspective of both disease pathophysiology and the potential for HDL therapies targeting this mechanism.

## HDL, Glucose Metabolism, and T2DM

T2DM, insulin resistance and glucose intolerance are associated with low HDL cholesterol levels (Ford et al., 2002; Drexel et al., 2005; Gatti et al., 2009). While there are clear mechanisms by which insulin resistance contributes to low HDL cholesterol, there is also evidence that low HDL may actually promote development of diabetes (Drew et al., 2012). Low HDL cholesterol levels predict development of T2DM in prediabetics (Haffner et al., 1990) and progression of glycemia in those with established T2DM (Waldman et al., 2014). With the recent recognition of the importance of HDL function above simple plasma HDL cholesterol concentration (Rohatgi et al., 2014), it is interesting to note that HDL cholesterol efflux capacity is inversely related to T2DM in the EPIC Norfolk study (Saleheen et al., 2015).

On the other hand, a recent Mendelian randomization study showed that genetically reduced HDL cholesterol does not associate with increased risk of T2DM (Haase et al., 2015). While this may reflect that the observational association is due to confounding and/or reverse causation, consideration must also be given to the fact that HDL cholesterol, while clinically accessible, does not accurately reflect HDL function and may not therefore be the best instrumental variable to test. As HDL function assays such as cholesterol efflux capacity become feasible in larger data sets, the hypothesis that HDL function may contribute causally to the risk of T2DM can be further explored using Mendelian approaches. In the meantime, mechanistic and intervention studies are uncovering links between HDL and glucose metabolism, which may have physiological and therapeutic relevance.

### Insulin Secretion

High-density lipoprotein has been implicated in the modulation of insulin secretion in cellular and animal studies with corroborating evidence from human intervention trials. The diverse functionality of HDL to combat cellular lipid accumulation, endoplasmic reticulum (ER) stress and apoptosis are three potential mechanisms that may preserve pancreatic beta cell function.

Pancreatic lipid accumulation and lipotoxicity have been well-documented to inhibit insulin production and secretion (Hao et al., 2007). We have shown that impairment of glucose-stimulated insulin secretion induced by oxidized low-density lipoprotein (LDL) can be countered by native HDL (50μg/mL) treatment (Drew et al., 2009). Further to this, a single dose of either wild-type ApoA-I (major protein component of HDL) or ApoA-I Milano was able to potentiate glucose-stimulated insulin secretion in high-fat fed mice (Stenkula et al., 2014). These effects on beta cell function may be mediated by the bioactive lipid sphingosine-1-phosphate (S1P), which is primarily carried within HDL particles and is known to independently promote glucose-stimulated insulin secretion (Cantrell Stanford et al., 2012).

At the signaling level the HDL transporters ATP-binding cassette, sub-family A, member 1 (ABCA1) and ATP-binding cassette, sub-family G, member 1 (ABCG1) have both been implicated in HDL-mediated effects on insulin secretion. Beta cell-specific deletion of the ABCA1 (Brunham et al., 2007) and ABCG1 (Sturek et al., 2010) transporters in mice impairs both cholesterol homeostasis and insulin secretion. Combined inactivation of ABCA1 and ABCG1 in beta cells increased intracellular cholesterol accumulation and induced beta cell inflammation, exacerbating the insulin secretory defect found in the single transporter knockout mice (Kruit et al., 2012). Furthermore, post-transcriptional suppression of ABCA1 via over expression of miR33a and miR145 in beta cells increased islet cholesterol levels and decreased glucose-stimulated insulin secretion (Wijesekara et al., 2012; Kang et al., 2013); effects that could be rescued by cholesterol depletion.

HDL may also influence insulin secretion via mechanisms other than cholesterol depletion, including via actions on insulin transcription (Fryirs et al., 2009; Cochran et al., 2014). At doses of up to 900μg/mL, lipid-free recombinant ApoA-I, ApoA-II and discoidal rHDL dose-dependently increased basal and glucose-stimulated insulin secretion in addition to insulin transcription (Fryirs et al., 2009). Whereas other experiments in MIN6 cells, as well as in islets isolated from mice and humans, found no evidence that HDL can directly enhance insulin production, basal or glucose-stimulated insulin secretion (Abderrahmani et al., 2007; Rutti et al., 2009).

The ability of HDL to inhibit ER stress-induced beta cell apoptosis could be another important mechanism by which HDL may ameliorate beta cell dysfunction (Petremand et al., 2009; Rutti et al., 2009). Bioactive lipids associated with HDL, such as S1P may also mediate anti-apoptotic effects on beta cells indirectly by interfering with the release of proapoptotic signals from non beta-cells within the pancreatic islet (Vollenweider et al., 2015). While it is more challenging to directly examine the effects of HDL on insulin secretion in humans, acute infusion of rHDL in patients with T2DM increased plasma insulin concentrations (Drew et al., 2009). Chronic HDL elevation induced by a CETP inhibitor in healthy individuals was also associated with an increase in postprandial plasma insulin and C-peptide concentrations (Siebel et al., 2013). Furthermore, plasma taken from these CETP inhibitor-treated individuals increased glucose-stimulated insulin secretion and cholesterol efflux from MIN6 beta cells relative to plasma from placebo-treated individuals. In this study, the actions of CETP inhibition on glucose-stimulated, but not basal insulin secretion suggests that HDL may be important in minimizing postprandial glucose excursions without raising resting insulin levels. Future clinical studies utilizing stable isotope tracers and minimal modeling techniques (Cobelli et al., 2014) are warranted to more directly characterize the effects of HDL on postprandial insulin secretion and glucose metabolism.

### Insulin-independent Glucose Uptake in Skeletal Muscle

**FIGURE 1:**
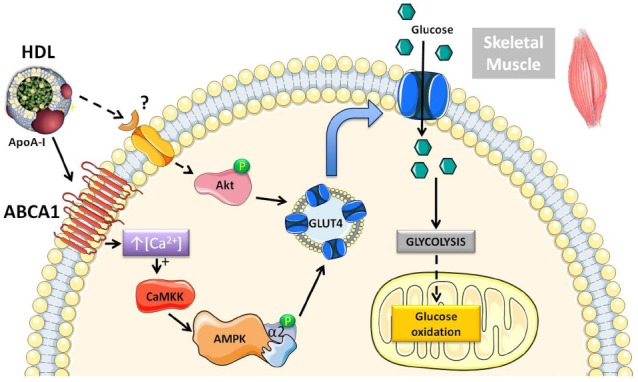
**Schematic diagram of the pathways activated by HDL in skeletal muscle that modulate glucose metabolism.** Both human and rodent studies have demonstrated that HDL can increase glucose uptake through an ABCA1/AMPK-dependent mechanism (Drew et al., 2009). Chronically elevating HDL levels pharmacologically or via genetic approaches in mice increases Akt phosphorylation, suggesting activation of an additional signaling pathway (Lehti et al., 2013). There is also more recent evidence in mouse muscle cells showing that HDL can directly stimulate anaerobic glycolysis and glucose oxidation.

We have previously shown that acute rHDL infusion can reduce blood glucose in individuals with T2DM (Drew et al., 2009). This reduction in glucose preceded an increase in plasma insulin, supporting the hypothesis that HDL may also mediate insulin-independent glucose uptake within skeletal muscle (Drew et al., 2009). This has been further investigated in primary human skeletal muscle cells and adipocytes where both HDL and ApoA-I promoted glucose uptake independently of insulin stimulation (Han et al., 2007; Drew et al., 2009; Zhang et al., 2011; Dalla-Riva et al., 2013). Figure 1 depicts the intracellular signaling pathways activated by HDL in skeletal muscle. HDL-associated ApoA-I binds to ABCA1 inducing intracellular Ca^2+^ influx and activation of calcium/calmodulin activated protein kinase kinase (CaMKK) (Drew et al., 2009). Subsequent activation and phosphorylation of AMP-activated protein kinase (AMPK) increases translocation of glucose transporter 4 (GLUT4) to the plasma membrane (Dalla-Riva et al., 2013) and facilitates glucose uptake.

Beyond glucose uptake, HDL has been shown to activate Akt in ApoA-I transgenic mice and directly enhance both glycolysis and glucose oxidation in mouse C2C12 muscle cells (Lehti et al., 2013). These insulin-independent effects of HDL on glucose metabolism, may actually account, at least partly, for *in vivo* effects of HDL on glucose disposal in the absence of a change in circulating insulin. Such effects may be mistakenly attributed to increased insulin sensitivity.

### Insulin Sensitivity and Glucose Homeostasis

Tissue lipid deposition and chronic inflammation associated with obesity, metabolic syndrome and T2DM establishes a functional deficit in skeletal muscle and the heart, impairing how these metabolically active tissues respond to insulin. The insulin resistance associated with lipid accumulation and a pro-inflammatory milieu promotes lipolysis, increasing circulating free fatty acids, thus perpetuating dyslipidemia and insulin resistance (Hotamisligil, 2006; Klop et al., 2013). It is still unclear whether it is the persistent hyperglycemia associated with T2DM that leads to the chronic inflammation typical of metabolic syndrome or whether the inflammatory response triggers an imbalance in glucose control and leads to insulin resistance. Either way via its effects on reverse cholesterol transport, functional HDL can remove lipids from peripheral cells and potentially ameliorate inflammation (Reviewed, in Navab et al., 2011). There is some cellular evidence that HDL elevation can increase insulin sensitivity in peripheral tissues (Carey et al., 2013).

Examining insulin sensitivity *in vivo* is more complex due to the fact that increased glucose disposal relative to insulin may not necessarily reflect increased insulin sensitivity. As discussed in the previous section, such observations may actually be explained by direct actions of HDL on glucose disposal by mechanisms which do not involve insulin. Nevertheless, there are two recent *in vivo* studies consistent with the cellular studies, which suggest that HDL/ApoA-I can increase peripheral insulin sensitivity. The first of these employed intraperitoneal glucose and insulin tolerance tests in high fat diet fed mice treated for 2–4 weeks with lipid-free ApoA-I (8 mg/kg body weight) and showed improved glucose and insulin tolerance compared to untreated mice on the same high fat diet (McGrath et al., 2014). The second raised HDL cholesterol levels using the CETP inhibitor torcetrapib in insulin resistant hamsters and found that torcetrapib increased insulin-stimulated glucose uptake in skeletal muscle, liver and the heart, improving glucose homeostasis (Briand et al., 2014).

There are a number of animal studies that implicate HDL and/or ApoA-I in the maintenance of glucose homeostasis, with considerable conjecture over whether HDL has a direct effect on insulin sensitivity. The relevance of these findings is generally not clear due to a lack of insulin tolerance (or more accurately insulin responsiveness) testing or hyperinsulinemic-euglycemic clamp studies to examine direct effects on insulin sensitivity. Definitive evidence for an effect of HDL on insulin sensitivity in humans is also lacking. However, HDL cholesterol elevation by CETP inhibition has been shown to improve glycemic control in the face of reduced plasma insulin in people with T2DM in the ILLUMINATE trial (Barter et al., 2011). As evidence for a role of HDL in glucose metabolism accumulates, clinical trials of HDL therapies are now including glycemic endpoints to further test clinical relevance.

## New Directions for HDL and Glucose Metabolism

The actions of HDL on glucose metabolism have potential benefit beyond vascular protection. The ability of HDL to stimulate tissue glucose uptake has implications for cellular metabolism in the brain, as well as for cell viability in other organs under ischemic/anoxic conditions where glucose becomes the predominant metabolic substrate. This latter mechanism may have particular relevance to ischemic vascular conditions, including coronary, cerebral and peripheral artery diseases.

### Ischemic Heart Disease

We have recently established that HDL increases insulin-independent glucose uptake in rat cardiomyocytes via an Akt signaling pathway (Heywood et al., 2014). In this cardiomyocyte model, the AMPK pathway involved in human skeletal muscle cell glucose uptake is not activated by HDL. It is known that plasma levels of HDL cholesterol are strongly associated with increased glucose disposal during the acute phase of myocardial infarction (Carvalho et al., 2012, 2013). However, the relevance of HDL-mediated glucose uptake to myocardial protection in human ischemic heart disease has not been established.

Insulin resistance may directly contribute to the increased risk of ischemic heart disease and poorer outcomes after infarction in patients with T2DM (Icks et al., 2014). It is plausible that HDL may improve cardiac function by directly increasing glucose uptake in addition to promoting efficient coupling of anaerobic glycolysis to glucose oxidation in the heart. This is particularly relevant in the setting of acute coronary syndromes where the ischemic myocardium is exposed to an acute ATP deficit, which can be ameliorated by targeting glucose metabolism immediately post-event in mice (Ussher et al., 2012; Masoud et al., 2014). In addition, the well-established vasodilatory effects of HDL via nitric oxide production (Mineo et al., 2003; Drew et al., 2004) may also enhance glucose uptake via microcirculatory vasodilatation allowing insulin and glucose access to nutritive vascular beds.

The emerging actions of HDL and its bioactive components on glucose metabolism in the myocardium may contribute to the demonstrated protective effects of HDL in the context of ischemia-reperfusion injury (Calabresi et al., 2003; Theilmeier et al., 2006; Herzog et al., 2010; Tao et al., 2010; Brulhart-Meynet et al., 2015). Such actions may be particularly important in the setting of T2DM, where non-insulin mediated glucose uptake could support tissue viability in the face of insulin resistance. Further investigations are required to determine the relevance of HDL-mediated effects on glucose metabolism and their relevance under ischemic conditions both in the presence and absence of insulin resistance. In this regard, novel reconstituted and recombinant HDLs have particular potential as anti-ischemic therapeutics in the setting of both acute coronary syndromes and coronary surgeries involving bypass procedures including coronary artery grafts (Kingwell and Chapman, 2013). Current trials with HDL infusion therapies (NCT02108262 and NCT02484378) will provide further insights into the actions of HDL in the context of acute coronary syndromes, though the potential role of glucose metabolism will not be discernible.

### Cerebrovascular Disease

For the same reasons that HDL-mediated effects on glucose metabolism may protect the ischemic myocardium, these actions may equally act in the brain to protect against transient ischemic attacks and stroke. In fact, the reliance of the brain on glucose for ATP production means that mechanisms controlling glucose uptake may be of even greater significance than in other organs. Even minor changes in cerebral blood flow have the potential to impact on neuronal viability via reduction in glucose supply. Indeed cerebral hypoperfusion and hypometabolism coincide in patients with dementia (Verfaillie et al., 2015).

The implications of reduced blood flow, and thus glucose supply to the brain, extend beyond acute stroke risk to Alzheimer’s disease, which has a significant vascular component (Di Marco et al., 2015). In this condition, cerebral blood flow is reduced by 10–30% in many brain regions, including those responsible for cognitive function and memory, with the extent of blood flow impairment correlating with disease severity (Jagust et al., 1997; Johnson et al., 1998; Hanyu et al., 2010; Kim et al., 2013). This reduction in cerebral blood flow is also detected prior to cognitive decline or amyloid accumulation. However, most relevant to this review, glucose uptake and utilization in the brain is also reduced in Alzheimer’s disease, mild cognitive impairment patients and transgenic animal models of Alzheimer’s disease (Herholz, 2010; Jagust et al., 2010; Shin et al., 2010; Nicolakakis and Hamel, 2011). Whether HDL can increase cerebral blood flow and glucose uptake in the central nervous system (CNS) is not currently known, but certainly worth exploring. Evidence to date has shown beneficial associations between brain health and HDL, but the contributing mechanisms have not been fully elucidated.

Given the well-established positive actions of HDL on vascular function, it is not surprising that several co-morbidities associated with increased Alzheimer’s disease risk are themselves associated with decreased HDL function (Riwanto and Landmesser, 2013). A role for HDL in glucose handling in the brain is an interesting candidate to partly explain the association of reduced circulating levels of HDL/ApoA-I with Alzheimer’s disease (Kuriyama et al., 1994; Kawano et al., 1995; Merched et al., 2000; Castano et al., 2006; Roher et al., 2009). Other mechanisms may relate to removal or breakdown of β-amyloid or reduction in its cytotoxicity (Paula-Lima et al., 2009; Lefterov et al., 2010). In this respect, ApoA-I may have actions analogous to apolipoprotein E (ApoE) where common genetic variants have directly opposing effects on β-amyloid deposition and clearance and therefore risk for Alzheimer’s disease (Corder et al., 1993). ApoE is the major apolipoprotein synthesized by astrocytes and microglia in the brain and forms lipoprotein particles that resemble HDLs found in plasma. Similarly to ApoA-I, ApoE is lipidated by ABCA1 (Pitas et al., 1987; LaDu et al., 1998; Fagan et al., 1999). Further work is required to understand the role of HDL and ApoA-I in brain lipid and glucose metabolism, both in the cerebral vasculature as well as neurons and glial cells and how this might relate to pathophysiology.

One important consideration for the potential application of HDL therapeutics to brain disorders is whether they can cross the blood-brain or blood-cerebral spinal fluid (CSF) barrier (Zlokovic, 2011). ApoA-I has been previously found in the CSF at relatively high levels (0.07–4 μg/mL or 0.1–0.5% plasma levels), (Koch et al., 2001) which is encouraging given the *in vitro* efficacy of ApoA-I at similar concentrations. More recently, Stukas et al. (2014a) have shown that intravenous injection of recombinant human ApoA-I specifically localizes to the choroid plexus. These data, along with *in vitro* primary cell culture experiments, suggest that plasma ApoA-I gains access to the CNS primarily by crossing the blood-CSF barrier via specific cellular mediated transport (Stukas et al., 2014a). The blood-CSF barrier is made up of highly fenestrated cuboidal epithelial cells within the choroid plexus (Lehtinen et al., 2013), which may explain how ApoA-I enters the CSF from the blood. This suggests that aside from the vasodilatory, anti-inflammatory and anti-oxidative actions of HDL/ApoA-I that could target cerebrovascular complications in Alzheimer’s disease, targeting brain-specific glucose metabolism may also be possible.

## Conclusion

There is now convincing evidence that HDL modulates glucose metabolism in multiple tissues. These actions have deepened our understanding of the pathophysiology of a variety of disease states associated with low or dysfunctional HDL. While there are still many unanswered questions relating to the underlying mechanisms and key HDL component(s) responsible for the metabolic effects, this opens up the possibility of targeting glucose metabolism with HDL therapeutics currently in development. Future preclinical investigations and clinical trials will determine the relevance of HDL-mediated modulation of glucose metabolism to both glycemic control as well as tissue glucose supply to vital organs including the heart and the brain, especially under ischemic conditions.

## Author Contributions

AS, SH, BK drafted, critically revised and approved the final version of this review.

### Conflict of Interest Statement

The authors declare that the research was conducted in the absence of any commercial or financial relationships that could be construed as a potential conflict of interest. BK has received product from CSL Behring and patient plasma from Hoffman La Roche both for investigator initiated clinical trials, with no associated research funding. She has also partnered with Resverlogix to fund an investigator-initiated clinical trial.

## Abbreviations

ABCA1: ATP-binding cassette sub-family A (ABC1), member 1
ABCG1: ATP-binding cassette sub-family G (ABC1), member 1
AMPK: AMP-activated protein kinase
ApoA-I: apolipoprotein A-I
ApoE: apolipoprotein E
CaMKK: calcium/calmodulin activated protein kinase
CETP: cholesteryl ester transfer protein
CNS: central nervous system
CSF: cerebral spinal fluid
ER: endoplasmic reticulum
GLUT4: glucose transporter 4
HDL: high-density lipoprotein
LDL: low-density lipoprotein
rHDL: reconstituted HDL
S1P: sphingosine-1-phosphate
T2DM: type 2 diabetes mellitus.
